# Looking ahead in early-phase trial design to improve the drug development process: examples in oncology

**DOI:** 10.1186/s12874-023-01979-5

**Published:** 2023-06-29

**Authors:** Alyssa M. Vanderbeek, Robert A. Redd, Steffen Ventz, Lorenzo Trippa

**Affiliations:** 1 Department of Data Science, Dana-Farber Cancer Institute, 450 Brookline Avenue, Boston, MA 02115 USA; 2 Unlearn.AI, San Francisco, CA USA; 3 Division of Biostatistics, University of Minnesota, Minneapolis, MN USA; 4 Harvard T.H. Chan School of Public Health, Boston, MA USA

**Keywords:** Drug development process, Clinical trial design, Simulation, Oncology

## Abstract

**Background:**

Clinical trial design must consider the specific resource constraints and overall goals of the drug development process (DDP); for example, in designing a phase I trial to evaluate the safety of a drug and recommend a dose for a subsequent phase II trial. Here, we focus on design considerations that involve the sequence of clinical trials, from early phase I to late phase III, that constitute the DDP.

**Methods:**

We discuss how stylized simulation models of clinical trials in an oncology DDP can quantify important relationships between early-phase trial designs and their consequences for the remaining phases of development. Simulations for three illustrative settings are presented, using stylized models of the DDP that mimic trial designs and decisions, such as the potential discontinuation of the DDP.

**Results:**

We describe: (1) the relationship between a phase II single-arm trial sample size and the likelihood of a positive result in a subsequent phase III confirmatory trial; (2) the impact of a phase I dose-finding design on the likelihood that the DDP will produce evidence of a safe and effective therapy; and (3) the impact of a phase II enrichment trial design on the operating characteristics of a subsequent phase III confirmatory trial.

**Conclusions:**

Stylized models of the DDP can support key decisions, such as the sample size, in the design of early-phase trials. Simulation models can be used to estimate performance metrics of the DDP under realistic scenarios; for example, the duration and the total number of patients enrolled. These estimates complement the evaluation of the operating characteristics of early-phase trial design, such as power or accuracy in selecting safe and effective dose levels.

**Supplementary Information:**

The online version contains supplementary material available at 10.1186/s12874-023-01979-5.

## Introduction

Most drug development processes (DDPs) in oncology consist of a series of early- to late-phase clinical trials. In most cases, each clinical study is designed based on a set of operating characteristics; for example, the power to detect treatment effects in a phase II study. The choice of the study design focuses primarily on the goals of a single clinical trial, often without estimates of the consequences of early-phase trials on later stages of the DDP. As we discuss in this article, early-phase trial designs can have a marked impact on the more time-consuming later stages of the DDP. Here, we use stylized simulation models to anticipate and describe relationships between early and late stages of drug development, with the goal of improving the design of early-phase clinical trials.

In precision medicine, the relationship between early and late stages of the DDP is particularly relevant; results from early phases, such as biomarker discovery and validation or identification of therapeutic targets and patient subpopulations, inform the design of subsequent trials in the DDP. Indeed, precision medicine involves the identification of subpopulations that benefit from experimental treatments [1, 2]. However, early-stage trial designs might be inadequate to accurately identify these subpopulations. As previously discussed in the literature [3], when early-phase trial designs are unable to capture treatment effect variations across subgroups, the power to detect treatment effects in later-stage trials might be reduced and the enrollment criteria may be suboptimal.

Innovations in the DDP are important to advance patient care; examples in clinical trial design include biomarker stratification and adaptive enrichment designs, as well as outcome-adaptive randomization, master protocols, platform trial designs, and the integration of real-world data [3–9]. These approaches have the potential to reduce the duration of the DDP, improve the accuracy of treatment effect estimates, and ultimately translate research into effective clinical care [2].

There are trade-offs between the operating characteristics of the DDP as a whole and each individual trial; these trade-offs may be difficult to quantify analytically. For example, the sample size of an early-phase trial to accurately identify subpopulations will influence the power of a subsequent phase III registration trial, as well as the costs and duration of the DDP. Simulations offer insights into the magnitude of these trade-offs and can support the design of trials throughout the DDP.

Important initiatives in oncology, from industry, academia, and regulatory agencies, recognize the importance of early-stage studies in drug development processes, and the potential for new strategies to improve study designs. For example, the FDA Optimus project (2023) focuses on the important transition from dose-selection studies centered on the estimation of the maximum tolerated dose, a standard goal for in the development of cytotoxic treatments, to designs that account for other outcomes beyond toxicities [10–12], such as tumor response. More generally, novel classes of treatments (e.g., immunotherapies [13]) present new challenges for the design of clinical trials.

Several aspects of the relationships between early-phase trial designs and later stages of the DDP have been discussed. For instance, data summaries from phase II trials, such as treatment effect estimates and confidence intervals, can inform the design of subsequent confirmatory studies [14, 15]. For the I-SPY 2 trial, Wang and Yee (2019) considered, using data observed throughout the phase II trial, the utility of predicting results of a subsequent confirmatory phase III trial to trigger interim and final decisions in early-stage trials, for example, the discontinuation of drug development [14]. De Ridder et al. (2005) investigate the impact of different distributions of pre-treatment prognostic characteristics in phase II and III trials on the likelihood of regulatory approval. Gotte et al. (2015) compared different strategies to choose early-phase trial sample sizes and evaluate how they impact on the power of subsequent phase III trials [16].

Finally, Conaway and Petroni (2019) present a simulation study evaluating the impact of early-phase trial design on the probability of regulatory approval [17]. Their work used interpretable metrics, such as the likelihood of identifying the maximum tolerated dose and the likelihood of successful regulatory approval.

We complement the existing body of work by considering stylized simulation models of consecutive clinical trials within oncology DDPs. Specifically, we show how such simulation models can be used to select design parameters (e.g., sample size of a dose-escalation study) for early-phase trials while accounting for potential downstream effects on subsequent trials and the DDP as a whole. We present three models:

1. A phase II single-arm trial and a subsequent phase III randomized controlled trial (RCT),
2. A phase I dose-selection trial and a subsequent phase II single-arm trial,
3. A phase II biomarker enrichment RCT [18] and a subsequent confirmatory phase III RCT.

The scenarios that we use to illustrate these simulation models are motivated by our previous work on the design of early stage trials in newly diagnosed glioblastoma [19–22], lung cancer [23], and breast cancer [24]. In all three examples, we discuss how the use of flexible simulation models of the DDP can be stylized to the clinical setting, support the design of early-phase trials. This approach complements the current practice of selecting sample sizes and other aspects of early-phase studies based on important trial-specific operating characteristics, such as power and study duration. R code for DDP simulations is provided as supplementary RMarkdown files.

## Methods

We considered three different DDP segments of two consecutive clinical trials. For each of the three examples, we proposed stylized models, where the results of the first trial (i.e., the final statistical analysis) determine whether to start the second trial in the segment or discontinue the DDP and influence the design of the second trial. A key decision in early-phase trial designs is the selection of the sample size, based on relevant trial-specific operating characteristics and performance metrics of the DDP segment, which are computed under plausible scenarios using simulations. In this paper, these performance metrics were tailored to each example. Table 1 defines the terms used frequently in the manuscript. We used the objective response rate (ORR; ${ORR}_{1}$ and ${ORR}_{0}$ to indicate the rates of experimental and standard of care treatments, respectively) as the primary endpoint in all three examples. Simulations were repeated 10,000 times and performed in R 3.6.0 (R Core Team) [25].

**Table 1:** Definitions

Term	Definition	Possible values	Example
Drug development process (DDP)	The consecutive series of early- to late-stage clinical trials that assess safety and efficacy of a new therapy	DDPs often consist of consecutive trials, from phase I trials to confirmatory studies	A new therapy is tested in a phase II trial that determines whether to advance the treatment to a phase III confirmatory trial
DDP segment	A subset of the DDP. In our illustration, the DDP segments consist of two consecutive clinical trials. The first of these trials is designed by the analyst and the results inform decisions about the second trial design	To simulate DDP segments, we specify scenarios and the statistical designs of two consecutive clinical trials. Simulations then provide a realistic representation of the DDP. The results from the first trial in the DDP segment inform relevant parameters, such as the sample size and enrollment criteria of the second study	A phase II trial with the primary aim of selecting the subpopulation that responds to the experimental treatment followed by a confirmatory trial that restricts enrollment only to the selected subpopulation
Performance metric	Operating characteristics of the DDP segment that illustrate the trade-off between costs, risks, time, and the likelihood of successfully developing the experimental therapy. Performance metrics across realistic scenarios can show whether study designs are aligned with the overall goals of the DDP and with the available resources. These metrics support the design of the first trial in the DDP segment based on its impact on the subsequent trial in the segment	Stylized simulations can be useful to estimate, under realistic scenarios, the numbers of patients to be enrolled in the DDP segment, the years to complete the segment, or the probability of a positive result of the DDP segment	The number of years necessary to complete a DDP segment

### Example 1: a phase II single-arm trial followed by a confirmatory phase III RCT

**Fig. 1:**
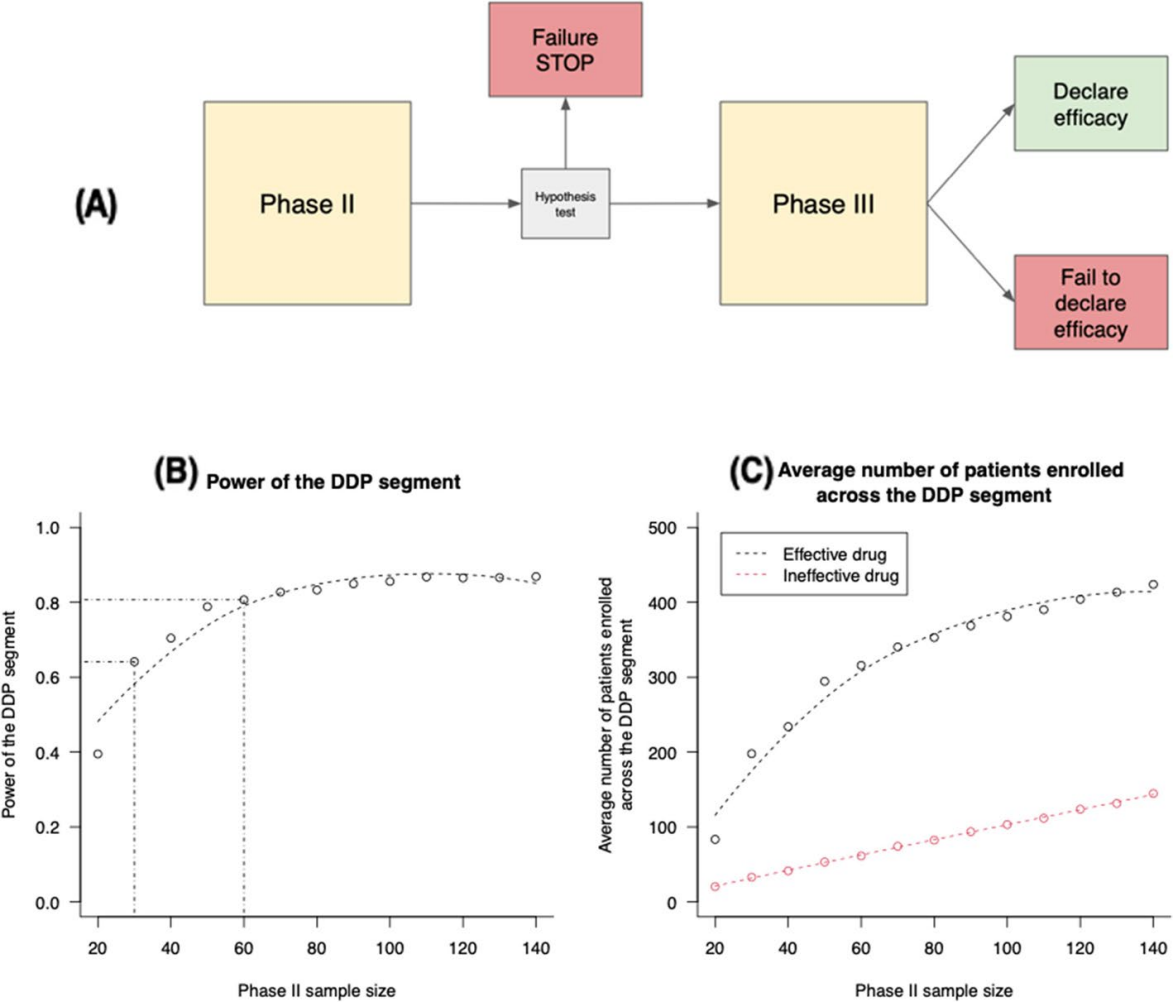
Example 1. **A** DDP segment: a phase II single-arm trial followed by a phase III RCT. The results of the phase II trial trigger the termination of the segment or continuation to phase III. **B** The probability that the phase II trial detects treatment effects and recommends a phase III trial that subsequently confirms the positive effect of the experimental treatment. **C** Average number of patients enrolled in the DDP segment (i.e., in the phase II and III trials) across simulations for an effective (black line) and ineffective (red line) therapy

This DDP segment comprises a single-arm phase II trial followed by a phase III RCT (Fig. 1). The analysis of this segment, and the simulation scenarios that we used, were motivated by the specific problem of selecting phase II trial designs for newly diagnosed glioblastoma [19, 20, 22] accounting for the impact of the design on the subsequent phases of the DDP.

The results from a single-arm phase II trial impact the decision to perform a phase III RCT or not and inform the design of the phase III trial. The phase II trial tests if the ORR of an experimental treatment is superior to the standard of care (SOC, null hypothesis ${{H}_{0}:ORR}_{1}\le 0.4$). In the DDP model, if the phase II trial rejects the null hypothesis based on an exact binomial test at a 0.05 significance level, then a phase III RCT is initiated. We considered different phase II sample sizes ${n}_{2}$. The sample size ${n}_{2}$ varies between 20 and 140 patients (Table 2), yielding a power between 70 and 99% with ORRs of 0.4 and 0.6 for the SOC and the experimental treatment. The phase III sample size is selected to attain a 90% power of rejecting the null hypothesis (${{H}_{0}:ORR}_{1}\le {ORR}_{0}$) using a Fisher’s exact test with a 0.05 type I error rate. The power calculation and the choice of the phase III sample size are based on the estimate ${\widehat{ORR}}_{1}$ from the phase II trial data and an historical estimate of ${ORR}_{0}$ equal to 0.4 for the SOC. Independent binary outcomes for trial participants are generated from binomial distributions with ${ORR}_{1}=0.6$ for the phase II, and ORRs equal to ${ORR}_{1}=0.6$ and ${ORR}_{0}=0.4$ for the phase III trial, conditional on treatment assignment.

**Table 2:** Description of the simulation models used to mimic DDP segments

**Parameter**	Example 1 Phase II trial followed by a phase III trial	Example 2 Phase I dose-finding trial followed by a phase II trial	Example 3 Phase II enrichment trial followed by a phase III RCT in the selected subpopulation
Sample size of the first trial	20–140 patients	10–80 patients	20–140 patients
SOC ORR	0.40	0.40	0.40
Hypothetical treatment effect used to choose the sample size for the second trial	Estimated from the phase II trial	0.20	Estimated from the phase II trial
Type I error rate of the second trial	0.05	0.05	0.05
Targeted power of the second trial	0.90	0.80	0.90
Sample size of the second trial	Computed using the results of the phase II trial. Maximum size: 400 patients	Calculated to achieve the desired power. Trial size fixed: 42 patients	Computed using the results of the phase II trial. Maximum size: 400 patients

We examined two performance metrics. First, we determined the probability that the phase II trial recommends a confirmatory phase III study that in turn demonstrates a treatment effect. We call this probability the *power of the DDP segment*. Second, we calculated the total number of patients enrolled in the DDP segment, i.e., in the phase II and phase III trials, and reported the mean across simulations.

### Example 2: a phase I dose-finding trial followed by a phase II trial

This DDP segment includes a phase I trial using the Continual Reassessment Method (CRM) design [26] followed by a phase II single-arm trial. This example and the simulation parameters were motivated by a phase I trial in non-small cell lung cancer that we designed at the Dana-Farber Cancer Institute [23].

**Fig. 2:**
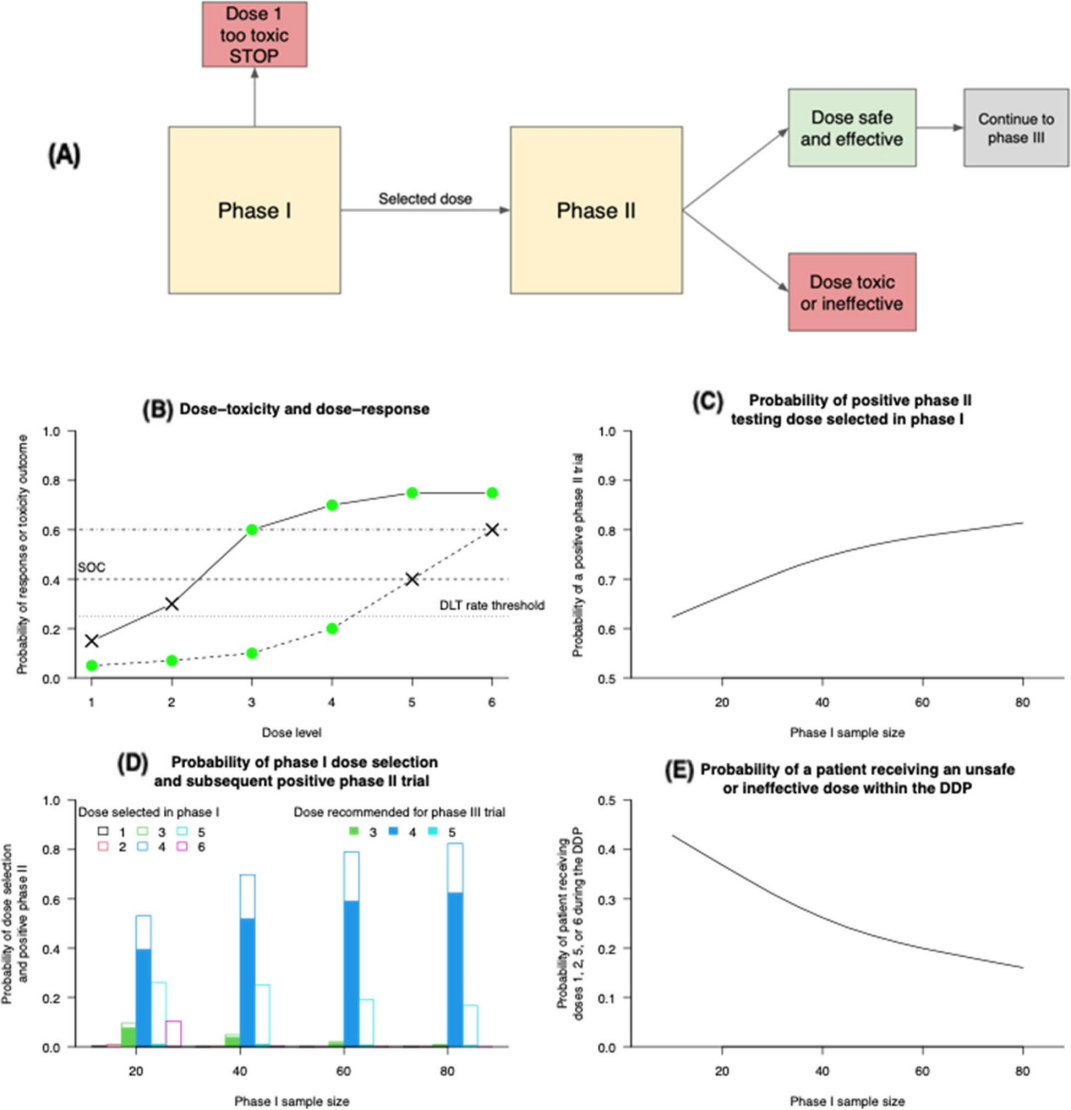
Example 2. **A** DDP segment: a phase I dose-finding study followed by a single-arm phase II trial. The DDP is discontinued after the phase I trial (i.e., treatment not recommended for phase II) if the lowest dose has a high toxicity estimate (> 0.25). The DDP segment is discontinued after the phase II trial if the trial does not detect efficacy (ORR ≤ 0.4). **B** The dose–response (solid line) and dose-toxicity (dashed line) relationship of the drug. Horizontal lines denote the ORR of the standard of care (dashed line), the experimental treatment (dotted-dashed line), and toxicity threshold (dotted line). Both the 3rd and 4th dose levels are safe (p ≤ 0.25) and effective (ORR > 0.4). **C** The power of the DDP segment (i.e., the probability that the phase I trial selects dose 3 or 4 and the subsequent phase II trial detects a treatment effect). **D** The probability that a dose is selected at completion of the phase I trial (clear bars). The panel also illustrates the probability that the phase II trial detects a treatment effect and recommends the drug for a phase III trial (solid subset of clear bars). **E** The probability that a patient enrolled in the DDP segment will be given an unsafe or ineffective dose

In the phase I CRM trial, both sequential dose assignments and the final dose selection are based on toxicity outcomes. The design targets the maximum tolerated dose (MTD), defined as the highest dose with probability of toxicity below a prespecified threshold (p). In our stylized model, the dose selected by the phase I trial is subsequently evaluated in a single-arm phase II trial. If a safe dose level is not identified by the phase I trial, then the DDP segment is discontinued (Fig. 2A). In the single-arm phase II trial, the experimental treatment is considered effective if it provides an improvement compared to a historical estimate for the SOC (${ORR}_{0}=0.4$, alternative hypothesis ${{H}_{1}:ORR}_{1}=0.6$) Toxicities are also evaluated at the end of the phase II trial, and the DDP is discontinued if the observed rate of adverse events in the phase II trial exceeds a pre-specified threshold.

The CRM trial evaluated six dose levels. We specified a toxicity threshold of p = 0.25, which is common in oncology [26]. The CRM trial was simulated under the dose–response/toxicity scenario displayed in Fig. 2B using the ‘dfcrm’ R package [27] with sample sizes ranging from ${n}_{1}$=10 to 80 patients. Toxicity outcomes in the phase I study at dose levels j = 1,…, 6 were generated according to the probabilities displayed in Fig. 2B. If the phase I study selected a dose ${j}^{*}$, then the binary outcomes of the patients in the phase II trial were generated according to the parameters of dose ${j}^{*}$ in Fig. 2B. The single-arm phase II trial with ${n}_{2}=42$ patients targeted 80% power (using an exact binomial test) with a significance level of 0.05 to test the hypotheses ${{H}_{0}:ORR}_{1}\le 0.4$ vs. ${{H}_{1}:ORR}_{1}=0.6$ (see Table 2). The sample size ${n}_{2}$ was fixed and independent of the selected dose level.

We assessed two performance metrics. First, we determined the power of the DDP segment, which here is the probability of a positive phase II trial combined with the selection of a safe and effective dose at the end of the phase I trial (i.e. probability of toxicity $P(DLT)\le p$ and $ORR>0.4$). Second, we determined the percentage of patients treated with an unsafe ($P\left(DLT\right)>p$) or ineffective ($ORR\le 0.4$) dose in the DDP segment. These are representative performance metrics; indeed, one could consider other metrics, such as the duration of the DDP segment.

Additionally, there is growing interest in early-stage trial designs that seeks to incorporate efficacy outcomes into dose-selection decisions, with the aim of recommending *optimal dose levels* (ODs) instead of MTDs. To provide an example (see Supplementary material for details) we explored a variation of the DDP segment that evaluates the same performance metrics (Figure S1). We considered a scenario with non-monotone dose–response relationship and replaced the CRM design with the Bayesian adaptive design of Zang et al. (2014) [12]. The design seeks to identify and recommend an OD level of an experimental agent instead of the MTD. The OD is defined as the dose-level j with the highest response rate (${ORR}_{j}$) among all dose-levels with acceptable toxicity [12].

### Example 3: a phase II enrichment RCT followed by a phase III RCT

**Fig. 3:**
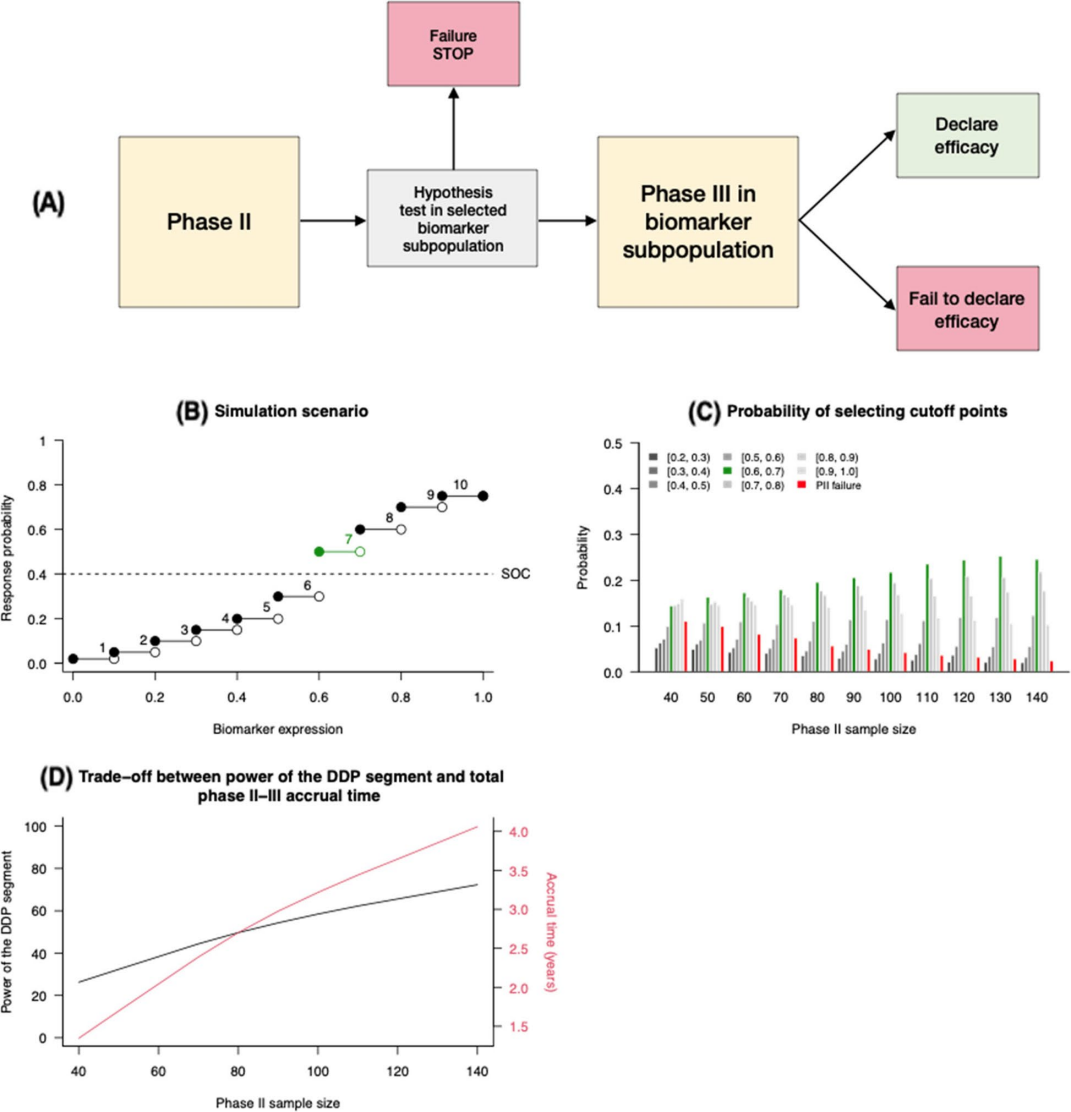
Example 3. **A** DDP segment: a phase II randomized population enrichment trial (Simon & Simon, 2013) followed by a phase III RCT in the selected subpopulation. The results of the phase II trial trigger termination of the DDP segment or continuation to a phase III trial. **B** Simulation scenario with a continuous biomarker. As biomarker levels increase, the probability of treatment response increases. The population with biomarker levels ≥ 0.6 benefits from the experimental treatment compared to the standard of care (SOC; dashed horizontal line), whereas patients with biomarker levels < 0.6 have better probability of response under the SOC. **C** The probabilities that each cutoff is selected at the end of the phase II trial. Selection of the optimal cutoff point (0.6) is shown in green. The probability of a negative result of the phase II trial, without evidence of treatment effects, is shown in red. **D** Power of the DDP segment

We considered a DDP segment consisting of a two-arm randomized phase II adaptive enrichment trial [18] followed by a phase III RCT (Fig. 3A). Several recent phase III RCTs (e.g., Sparano et al., 2018 [27]) evaluated experimental treatments in subpopulations that were previously identified in early-phase clinical studies [28–30].

In this DDP segment, the phase II trial design considers a continuous biomarker with the goal of determining a cutoff value that identifies the subpopulation of patients that benefit from the experimental treatment. If the phase II trial identifies a treatment effect in a subpopulation [18], then a phase III RCT is conducted. Eligibility for the phase III trial is limited to the subpopulation selected by the phase II study.

In the phase II trial population, the biomarker was uniformly distributed between 0 and 1. We considered nine candidate cutoff points starting at 0.1 up to 0.9 with increments of 0.1 (Fig. 3B). The trial had three accrual periods and two interim analyses (IAs); 50% of the patients were enrolled in stage 1, 25% in stage 2, and 25% in stage 3. The outcomes for patients in the experimental arm, given their biomarker value were generated according to the probabilities ${ORR}_{1}(x)$ displayed in Fig. 3B. The binary outcomes for the SOC arm were generated with ${ORR}_{0}(x)=0.4$, without variation across biomarker values. The phase II enrichment trial tests the null hypothesis ${{H}_{0}:ORR}_{1}(x)\le {ORR}_{0}(x)$ for all values of x between 0 and 1 using McNemar’s test (Simon and Simon 2013). We considered different phase II trial sample sizes, which varied from ${n}_{2}=20$ to 140 patients (Fig. 3).

The phase III sample size ${n}_{3}$ targeted a 90% power at a 0.05 significance level based on a Fisher’s exact test, with a maximum of 400 patients. The sample size varied across simulations, based on the estimated ORRs for the experimental and SOC treatments in the selected subpopulation, as reported by the phase II enrichment trial, for the power calculation. Outcomes were simulated by the same mechanism as in phase II, where the biomarker value is uniformly distributed between the cut-off selected by the phase II trial and one.

We examined two performance metrics of the DDP segment. First, we determined the power of the DDP segment, i.e. the probability that, for an effective drug, the phase II trial selects a cutoff point and rejects ${H}_{0}$ (i.e., the null hypothesis that all patients don’t benefit from the experimental treatment) and that the phase III trial confirms the result. Second, we evaluated the duration of the DDP segment under realistic assumptions about enrollment rates.

## Results

### ***Example 1***

The power of the DDP segment, as expected, was considerably reduced when we compared (i) a phase II design (the first component of the DDP segment) planned to achieve 80% power for our hypothetical treatment effect (${{H}_{1}:ORR}_{1}=0.6$) without considering the subsequent phase III confirmatory trial, and (ii) a trial design with a sample size that targets 80% power of the DDP segment (Fig. 1B). In the first case, the resulting sample size of the phase II trial was 30 patients. For the hypothetical treatment effect, with this phase II sample size, the *power of the DDP segment* was below 65%. Conversely, planning the phase II trial with the support of a simulation model that includes both studies in the DDP segment (target: 80% power of the DDP segment), the resulting phase II study had a sample size of 60 patients. This translated into a ~ 90% power of the phase II trial. This increase in power of the DDP segment was associated with an increase in average total sample size of the segment from 225 to 325 patients (Fig. 1B). Moreover, the additional power gained by enrolling more patients in phase II begins to increase at a substantially lower rate for sample sizes above ${n}_{2}=80$. For example, the DDP power increases from approximately 85% to 88% by increasing ${n}_{2}$ from 80 to 110 (Fig. 1B).

### ***Example 2***

Of the six doses considered in the phase I CRM trial, only dose levels 3 and 4 met the criteria for safety and efficacy. The other levels presented low ORR (doses 1 and 2) or were toxic (doses 5 and 6; Fig. 2B). Comparing performance metrics, a single-arm phase I trial with 20 patients was associated with 65% power of the DDP segment (i.e., probability of selecting dose 3 or 4 in the phase I study and detecting efficacy in the subsequent phase II trial; Fig. 2C). This sample size was also associated with a high probability that the phase II trial would evaluate and expose patients to ineffective or toxic doses (Fig. 2D-E). In contrast, enrolling 60 patients in the phase I trial provides 80% probability of selecting either dose 3 or 4 (Fig. 2D) and increases the power of the DDP segment to 75% (Fig. 2C).

When the relationship between dose level and response is non-monotone, phase I designs that identify ODs instead of MTDs can improve the power of the DDP segment and reduce the average number of toxicity events (Figures S1 and S2).

### ***Example 3***

In the third DDP segment, as the sample size of the phase II enrichment trial increased, the optimal biomarker cutoff point (0.6) was selected with increasing accuracy (Fig. 3C) and the probability of a negative phase II trial result that terminate the DDP decreased (Fig. 3C). This in turn led to a higher probability that the DDP segment has a positive result in the phase III trial (Fig. 3D). For example, if the phase II trial enrolled 40 patients, then the trial results recommend discontinuing the DDP with a probability of more than 10%. Additionally, with this sample size, cutoff points of 0.6 or greater were selected with approximately equal probabilities. In other words, with high probability the phase III trials enroll only a subset of patients that benefits from the experimental treatment. With this sample size, we estimated a 30% power of the DDP segment (i.e., detecting efficacy in the phase III trial; Fig. 3D). By contrast, enrolling 130 patients into the phase II enrichment trial increased the power of the DDP segment to 70%. There is a trade-off between the power of the DDP segment and the time necessary to complete the segment. Large phase II sample sizes increase the DPP power, but they also tend to extend substantially the duration of the DDP segment (Fig. 3D).

## Discussion

In this work, we (1) demonstrated the impact of early-phase designs and their parameters, such as sample size, on the operating characteristics and potential results of subsequent late-phase trials; (2) illustrated that simulation models are flexible, useful, and customizable to evaluate the relationships between early-phase trials and operating characteristics of the DDP; and (3) provided examples to facilitate the use of DDP simulation models in planning future early-phase trials in oncology.

The simulation-based approach that we proposed is applicable to trial designs and DDPs with various primary outcomes, including outcomes beyond responses (as considered in our examples). We use ORR in all phases for simplicity and simultaneously emphasize that the simulation-based framework that we proposed allows investigators to consider these designs and DDPs and compare early-trial designs with different primary outcomes. Comparing ORRs is one way to evaluate an experimental treatment; early-stage trial designs to evaluate a specific experimental treatment might use ORR, PFS, OS, or other novel measures as primary outcomes [31, 32]. The relative merits and weaknesses of these primary outcomes have been discussed in the oncology literature and vary substantially across patient populations, classes of treatments, and phases of drug development [33, 34].

In general, the choice of the primary endpoint should be tailored to the oncology trial and consider differences in study-specific aims across trials, strategies of different DDPs, and patient populations. For a DDP with ORR as the primary endpoint (or other binary endpoints) in phase II and OS in later-phase trials, then scenarios would need to be specified accounting for data and meta-analyses that allow the analyst to express realistic scenarios and explore operating characteristics of the DDP. Here, OS and PFS data can be generated concurrently for the phase II trial that utilizes ORR as primary outcomes, and these generated outcomes can be useful for designing the subsequent phase III study. This type of DDP segment involves some considerations. First, in the literature, discordant treatment effects have been reported between ORR and OS. Second, if the relationship between ORR and OS outcomes is well-informed, OS data generated concurrently in the phase II trial can be useful for designing the subsequent confirmatory study.

In Example 1, we examined the relationship between the sample size of a phase II trial and the likelihood that a subsequent phase III trial would demonstrate improved outcomes for the experimental treatment compared to the SOC. This example illustrated how simulations and analyses restricted to a single clinical trial versus extended perspectives that account for subsequent studies in the DDP can lead to markedly different decisions on key aspects of the early-phase design such as the sample size.

In Example 2, we considered a phase I dose-finding trial designed to identify the highest tolerated dose (MTD) to be evaluated in a subsequent phase II study. The simulation model quantified the extent to which increasing the phase I sample size would affect the likelihood of a positive result at completion of the DDP segment. Stylized models of DDP segments can be used to compare early-phase trial designs (e.g., 3 + 3 design [35], CRM design [26], Bayesian Optimal Interval (BOIN) design [36], EffTox design [37], etc.) under plausible scenarios. These comparisons can include phase I designs that utilize both toxicity and response outcomes (see the Supplementary Material for one example).

In Example 3, we considered a phase II adaptive enrichment design [18]. Our model of the DDP segment quantified the extent to which the phase II sample size contributes to the power of the DDP segment. Our stylized simulations evaluate the accuracy of the DDP segment in identifying the subpopulation that benefits from the experimental therapy. Simulations can also be used to explore implications of the phase II trial design on the DDP costs and duration.

In all our examples, the outlined performance metrics of the DDP segments are not intended to constitute an exhaustive assessment. Rather, they exemplify the trade-offs between sample size, trial conclusions, accuracy, and time necessary to evaluate treatments in DDP segments. One limitation of this work is that our DDP segments contain only two consecutive trials. However, in certain cases it may be useful to consider the complete DDP, from the trial that the investigators are designing onward. Additionally, in our examples, the sample size of the second clinical trial is selected using only a point estimate of the treatment effects from the first clinical study. In several cases it is appropriate to account also for the variance and confidence interval of this estimate.

Several contributions point at the importance of adequate sample sizes in early-phase trials [16, 38]. For instance, unrealistic expectations of large treatment effects have been associated with poor sample size decisions and trial designs [38]. We showed, through simple simulation models, how the sample sizes of early-phase trials can impact on the resource requirement and the probability of positive findings of the DDP segments. For example, in our first DDP segment, a phase II sample size of 30 patients achieved trial power equal to 80%, and the probability of a positive finding at completion of the DDP segment was only 65%, while increasing the phase II sample size to 60 patients yields 80% probability of a successful DDP segment. Moreover, significance levels different from the ubiquitous 5% level can be explored. Realistic and well-justified treatment effects hypotheses and scenarios are necessary for standard power calculations as well as for simulation-based analyses of DDPs to support decisions about key parameters early-stage trial designs.

Simulation reports used for the purpose of supporting trial designs need to balance (i) the use of comprehensive and plausible sets of scenarios (e.g., potential dose-toxicity and dose-efficacy curves) and (ii) multiple operating characteristics with (iii) concise summaries of the analysis to compare candidate trial designs. The Food and Drug Administration (FDA) Model-Informed Drug Development (MIDD) guidance (2021) acknowledges that “*when successfully applied, MIDD approaches can improve clinical trial efficiency, increase the probability of regulatory success, and optimize drug dosing/therapeutic individualization*” [39]. Simulation models have been useful in supporting decision-making in drug development. Data and results from meta-analyses and previous studies as well as information on regulatory standards and recruitment rates are fundamental in the development of useful simulation models of DDPs.

DDP models can support decision-making on early-phase trial designs by improving comparisons of candidate trial designs and taking into consideration the impact of early-stage trials on subsequent trials in the DDP. We showed here that simple and stylized simulations of DDP segments can effectively complement the use of standard operating characteristics (e.g., power and duration of a clinical trial). Ultimately, better decision-making on trial designs improves the efficiency of the DDP a whole as and accelerates the translation of clinical trial findings into clinical care.

## Conclusions

We discussed how stylized simulation models of clinical trials in an oncology DDP can quantify important relationships between early-phase trial designs and their consequences for the remaining phases of the DDP. These models can be used to estimate and compare performance metrics of the DDP under realistic scenarios; for example, the duration and the total number of patients enrolled. These estimates complement the evaluation of operating characteristics of early-phase trial design, such as power and sample size.

## Supplementary Information

**Additional file 1.**

**Additional file 2.**

## Data Availability

This is a simulation study and as such, all data used in this study is generated using the parameters and procedures described in the main text. This generated data is meant to mimic future clinical trials, but neither used nor includes real patient data. The datasets used and/or analysed during the current study are available from the corresponding author upon reasonable request.

## Abbreviations

BOIN: Bayesian Optimal Interval
CRM: Continual reassessment methods
DDP: Drug development process
DLT: Dose-limiting toxicity
FDA: Food and Drug Administration
IA: Interim analysis
MIDD: Model-Informed Drug Development
MTD: Maximum tolerated dose
OD: Optimal dose
ORR: Objective response rate
RCT: Randomized controlled trial
SOC: Standard of care
