# Supplementary material for: Looking ahead in early-phase trial design to improve the drug development process: examples in oncology
Source: BMC Med Res Methodol. 2023 Jun 29;23:151. doi: 10.1186/s12874-023-01979-5 (PMC10308797; doi:10.1186/s12874-023-01979-5)
Supplement: Supplementary file 2 — Additional file 2. [file 12874_2023_1979_MOESM2_ESM.docx]

**Supplementary: “Looking Ahead in Early-Phase Trial Design to Improve the Drug Development Process: Examples in Oncology”**

Alyssa M Vanderbeek, MS^1,2^*; Robert A Redd, MS^1^*; Steffen Ventz, PhD^3^; Lorenzo Trippa, PhD^1,4^

*Co-first authors

*^1^Department of Data Science, Dana-Farber Cancer Institute, Boston, MA, USA;*

*^2^Unlearn.AI, San Francisco, CA, USA;*

*^3^Division of Biostatistics, University of Minnesota, Minneapolis, MN, USA;*

*^4^Harvard T.H. Chan School of Public Health, Boston, MA, USA*

**Example 2b: : a phase I Bayesian adaptive dose-finding trial followed by a phase II trial**

We implemented a DDP segment that consists of

(i) a dose-finding trial that seeks to identify an optimal dose (OD) for a new experimental treatment and

(ii) a subsequent trial that tests the efficacy of the experimental treatment (at the OD recommended by the dose-finding trial) compared to the standard of care therapy.

For (i), we considered the Bayesian adaptive design of Zang et al. [Clinical Trials 2014, 11:319-327] to identify ODs. The use of this design is in part motivated by the availability of software to implement simulations in R, and the possibility to plug-in the software in our pipeline to study the DDP segment. This was not possible for other candidate designs. The OD in the Zang et al. design is defined as the dose level with the highest response rate (${ORR}_{j}$) among all dose levels with acceptable toxicity (i.e., dose levels j with toxicity probability $p_{T,j}\leq\varphi$).

The design sequentially enrolls cohorts of five patients in T stages (we considered T=4, 5, …, or 28 stages in our simulations) to one of the six dose levels. At each stage t=1,..., T, the design first identifies all dose-levels $1\leq j\leq6$with acceptable toxicity level, i.e., dose levels with posterior probability $P(p_{T,j}>\varphi|\text{data by stage }t)<c_{T}$ (we used default values$\varphi=0.3, c_{T}=0$ in our simulations). If no dose satisfies the requirement then, the trial stops. Otherwise, the design estimates the OD, given the current data, by selecting the dose level with the highest estimated response rate among all safe dose levels. It then assigns the next cohort of five patients to the estimated OD level.

If the trial does not terminate early (i.e., before the enrollment of $5T$patients), then, at completion of the study after the enrollment of $n_{1}=5T$ patients (we considered n1=20, 25, …, or 140 in our simulations), the design provides a final estimate of the OD, given the available data at completion of the study.

The subsequent single-arm phase II study tests ORR of the experimental treatment at the recommended phase I OD level against the historical ORR of the SOC ${H_{0}:ORR}_{1}\leq0.4$. This design is identical to the one used in Example 2.

Our supplementary simulations (Figures S1 and S2) showed that, for experimental treatments with non-monotone dose-response curves, the implementation of a phase I design that identifies the OD instead the MTD can improve the power of the DDP segment and reduce the average number of toxicity events for patients in the DDP segment. This also highlights the importance of selecting phase I trial designs that fit well with existing knowledge about the experimental therapy (e.g., from meta-analyses of drug classes and pre-clinical studies) and the disease.

Figures S1: DDP segment: a phase I dose-finding study (using the Bayesian adaptive design of Zang et al. (2014)) followed by a single-arm phase II trial. (B) The dose-response (solid line) and dose-toxicity (dashed line) relationship of the drug. Horizontal lines denote the ORR of the standard of care (dashed line), the experimental treatment (dotted-dashed line), and toxicity threshold (dotted line). (C) The power of the DDP segment (i.e., the probability that the phase I trial selects dose 3 or 4 and the subsequent phase II trial detects a treatment effect). (D) The probability that a dose is selected at completion of the phase I trial (clear bars). The panel also illustrates the probability that the phase II trial detects a treatment effect and recommends the drug for a phase III trial (solid subset of clear bars).

Figures S2: DDP segment: a phase I dose-finding study (using the CRM design) followed by a single-arm phase II trial. (B) The dose-response (solid line) and dose-toxicity (dashed line) relationship of the drug. Horizontal lines denote the ORR of the standard of care (dashed line), the experimental treatment (dotted-dashed line), and toxicity threshold (dotted line). (C) The power of the DDP segment. (D) The probability that a dose is selected at completion of the phase I trial (clear bars). The panel also illustrates the probability that the phase II trial detects a treatment effect and recommends the drug for a phase III trial (solid subset of clear bars).

Table S1: Simulation parameters for all examples.

**References**

Zang Y, Lee JJ, Yuan Y. Adaptive designs for identifying optimal biological dose for molecularly targeted agents. Clin Trials. 2014;11(3):319-327.
